# 188. Visualizing the Impact of a Wedding Leading to COVID-19 Outbreaks in Healthcare Settings, Washington State, July – August 2020

**DOI:** 10.1093/ofid/ofab466.188

**Published:** 2021-12-04

**Authors:** Audrey Brezak, Anna Unutzer, James W Lewis, Shauna Clark, Jessica Ferro, Siri Bliesner, Julie Loughran, Vance Kawakami, Ruth Deya, Rhodah Makayoto, Em Maier, Holly Whitney, Elyse Bevers, Allison A Templeton, Bonnie Burlingham, Larissa Lewis, Sara T Podczervinski

**Affiliations:** 1 Washington State Department of Health, BELLINGHAM, Washington; 2 Public Health Seattle & King County, Seattle, Washington; 3 Public Health - Seattle & King County, Bellevue, Washington; 4 Public Health Seattle and King County, Seattle, Washington; 5 Public Health – Seattle & King County, Seattle, Washington; 6 Public Health- Seattle & King County, Seattle, Washington; 7 WA Dept of Health, Seattle, Washington; 8 Washington State Deparment of Health, Olympia, Washington

## Abstract

**Background:**

Large social gatherings during the COVID-19 pandemic have been linked to extensive community transmission. Healthcare workers (HCW) that engage in these social gatherings pose a risk to the vulnerable patients they serve. Public Health—Seattle & King County identified a COVID-19 outbreak associated with a wedding in July 2020 when the 14-day incidence rate was 105 cases per 100,000 residents. HCW who attended the wedding were subsequently linked to 45 outbreaks in healthcare settings across three counties in the next month.

**Methods:**

COVID-19 case interview data was used to identify HCW cases who reported the wedding as their exposure event. The Washington Disease Reporting System (WDRS), the state database in which COVID-19 cases and epi-linkages are tracked, was queried to identify healthcare outbreaks linked to the HCW wedding-attendee cases and the HCW that they infected. NodeXL was used to visualize the resulting chains of wedding-associated healthcare transmission using a Harel-Koren Fast Multiscale layout where the network visualization’s directed arrows represent putative links and direction of transmission. Numbers of associated settings, cases, and deaths were calculated.

**Results:**

Seven HCW wedding attendees were linked to outbreaks in healthcare facilities that they worked at while infectious; HCWs linked to as many as six subsequent healthcare outbreaks. In total, the wedding was connected to 45 healthcare facilities: adult family homes (N=1), hospitals (N=1), supported living agencies (N=7) and associated group homes (N=38), assisted living (N=1), home health services (N=1), behavioral health (N=2), and rehab centers (N=1). Across the settings, 277 cases were identified, including 15 deaths.

**Conclusion:**

A series of COVID-19 healthcare outbreaks was traced back to a wedding. Cases worked in multiple homes, agencies, and other healthcare settings which likely facilitated rapid and wide transmission; the structure of these healthcare settings often do not facilitate a single job providing enough hours and income to support an individual. In terms of public health learnings, addressing these outbreaks require effective contact tracing, multijurisdictional coordination, and for supported living, interventions need to be applied across households sharing staff.

**Disclosures:**

**All Authors**: No reported disclosures

